# The Influence of the Technique of Surfactant Administration (LISA *vs* INSURE) on the Outcomes of Respiratory Distress Syndrome Treatment in Preterm Infants

**DOI:** 10.34763/devperiodmed.20192303.163171

**Published:** 2019-10-27

**Authors:** Urszula Kaniewska, Ewa Gulczyńska

**Affiliations:** 1 Department of Neonatology, Polish Mother’s Memorial Hospital Research Institute, Łódź, Poland

**Keywords:** LISA, INSURE, respiratory distress syndrome (RDS), bronchopulmonary dysplasia (BPD), surfactant, LISA, INSURE, zespół zaburzeń oddychania, dysplazja oskrzelowo-płucna, surfaktant

## Abstract

Intratracheal administration of exogenous surfactant is a well-established therapy for respiratory distress syndrome in preterm infants. The two preferred methods for respiratory support in neonates that contribute to limiting the risk of lung damage associated with mechanical ventilation include nCPAP and non-invasive ventilation. The increasing popularity of surfactant administration techniques is due to the fact they reduce the time of mechanical ventilation until this medication is administered. In some cases a short period of mechanical ventilation follows (INSURE: INtubation-SURfactant-Extubation). There are also methods that make it possible to completely avoid intubation and help maintain spontaneous breathing during surfactant administration (LISA: Less Invasive Surfactant Administration, MIST: Minimal Invasive Surfactant Therapy).

**Aim:**

To analyze treatment outcomes in preterm infants who suffer from respiratory distress syndrome and require exogenous surfactant administration depending on the technique used: LISA vs INSURE.

**Material and methods:**

The present retrospective analysis included 129 infants born at a gestational age of between 24 and 33 weeks who were hospitalized in the Neonatology Department in the years 2014-2016, were administered surfactant and remained on non-invasive ventilation. All the subjects received only proractant alfa. Both study groups: LISA (n=83) and INSURE (n=46) were analyzed in terms of respiratory distress treatment outcomes and the presence of complications of prematurity.

**Results:**

There were no significant differences in patient characteristics between the two study groups (LISA vs INSURE: mean birth body weight was 1210g vs 1275 g, respectively; mean gestational age at birth was 30 weeks vs 29 6/7 weeks, respectively). The comparison of respiratory support method and FiO2 concentration within the first 72 hours after surfactant administration showed no significant differences between the groups. Similarly, respiratory outcomes did not significantly differ between the LISA and INSURE groups and were: the need for intubation ☒ 42.2% vs 32.6%, p=0.201, duration of mechanical ventilation – median days 0 vs 0, p=0.377, duration of nCPAP – median days 5 vs 5, p=0.379, duration of oxygen supplementation – median days 1 vs 1, p=0.555, and the incidence of bronchopulmonary dysplasia – 28.9% vs 23.9%, p=0.506. Also, the incidence of complications was similar in both study groups.

**Conclusions:**

Our retrospective analysis of preliminary outcomes of surfactant administration involving the use of the LISA technique showed no statistically significant differences as compared with the INSURE method. The randomized, prospective study that is currently being conducted at our Neonatology Department and includes biochemical markers of lung damage, will bring more objective data on the safety and effectiveness of both surfactant administration techniques (LISA vs INSURE).

## Streszczenie

Dotchawicze podanie surfaktantu egzogennego jest standardową metodą leczenia zespołu zaburzeń oddychania u noworodków urodzonych przedwcześnie. Jednocześnie preferowanym obecnie sposobem wsparcia oddechu u noworodków jest nCPAP lub wentylacja nieinwazyjna, które pozwalają na zmniejszenie ryzyka uszkodzenia płuc związanego z wentylacją mechaniczną. Stąd coraz częściej stosowane są metody podaży surfaktantu, ograniczające wentylację mechaniczną tylko do czasu podania surfaktantu i ew. krótko po podaniu leku (INSURE; intubacja-surfaktant-ekstubacja) lub pozwalające na całkowitą rezygnację z intubacji i zachowanie spontanicznego oddechu w czasie podaży leku metodą LISA/MIST (Less Invasive Surfaktant Administration, Minimal Invasive Surfactant Administration).

**Cel:**

Analiza wyników leczenia noworodków urodzonych przedwcześnie z zespołem zaburzeń oddychania, wymagających podaży surfaktantu egzogennego, w zależności od techniki podania leku: LISA vs INSURE.

**Materiał:**

i metody: Badaniem retrospektywnym objęto 129 noworodków o dojrzałości 24-33 tyg. ciąży, leczonych w Klinice Neonatologii w latach 2014-2016, które otrzymały surfaktant pozostając na wsparciu nieinwazyjnym. Dzieci poddane analizie otrzymywały wyłącznie proractant alfa. Badane grupy noworodków: LISA (n-83) oraz INSURE (n-46) analizowano pod kątem wyników leczenia niewydolności oddechowej oraz powikłań charakterystycznych dla wcześniactwa.

**Wyniki:**

Analizowane grupy noworodków nie różniły się pod względem danych demograficznych (średnia m. ciała 1210 g vs 1275 g; średnia dojrzałość płodowa 30 vs 29 6/7 tyg. ciąży odpowiednio LISA vs INSURE). Porównując tryb wsparcia oddechowego oraz stężenie FiO2 w pierwszych 72 hod podania surfaktantu nie wykazano różnic między grupami. Analizując wyniki oddechowe nie stwierdzono różnic istotnych statystycznie między grupami, LISA vs INSURE: intubacja (42,2% vs 32,6%, p=0,201), liczby dni wentylacji mechanicznej (mediana 0 vs 0, p=0,377), liczby dni wsparcia oddechu metodą nCPAP (mediana 5 vs 5, p=0,379), dni tlenoterapii biernej (mediana 1vs 1, p=0,555) oraz częstość występowania dysplazji oskrzelowo-płucnej (28,9% vs 23,9%, p=0,506). Również dla powikłań charakterystycznych dla wcześniactwa nie obserwowano istotnych różnic.

**Wnioski:**

W prezentowanej retrospektywnej analizie wstępnych doświadczeń z podażą surfaktantu metodą LISA nie wykazano istotnych statystycznie różnic w stosunku do grupy noworodków otrzymujących surfaktant metodą INSURE. Aktualnie prowadzone w Klinice Neonatologii prospektywne badanie z randomizacją, w którym oznaczane będą także biochemiczne markery uszkodzenia płuc, pozwoli na uzyskanie zobiektywizowanych wyników dotyczących bezpieczeństwa i efektywności obu metod podaży surfaktantu (LISA vs INSURE).

## Introduction

Continuous improvements in perinatal care lead to an increase of the survival rates in the population of preterm infants. The methods of reducing lung damage in these patients are, however, still limited, and show no spectacular influence on the risk of bronchopulmonary dysplasia (BPD). Reducing or avoiding the need for mechanical ventilation by means of implementing noninvasive respiratory support techniques results in the improvement of respiratory outcomes. A key element of non-invasive ventilation is the time of exogenous surfactant administration. The present study compares two non-invasive methods of surfactant administration: LISA and INSURE.

The INSURE method was first described by Henrik Verder et al. in 1994 [1]. Since that time, it has been the subject of over 500 articles. On the other hand, the LISA method, reported in 1992 by the same author [2], did not at first attract considerable interest. It was not until the next decade (2001) that the LISA method was reintroduced and described by A. Kribs et al. from Cologne, who is now widely considered a pioneer of the LISA method.

The INSURE method involves a certain sequence of actions: intubation (IN), surfactant administration (SUR) and extubation (E). For an experienced physician working in an intensive care unit it is a standard, relatively easy procedure. On the other hand, during LISA, the newborn keeps breathing spontaneously with nasal support (nCPAP). A thin catheter is placed in the trachea during laryngoscopy with (or without) the use of Magill forceps and the surfactant is then installed in a rapid bolus. This process requires the correlation of several actions, which can be achieved only through training and practice. The implementation of LISA technique in the Neonatology Department was started in 2014. The procedure has been gradually introduced to physicians working in the Intensive Care Unit, including residents.

## Aim

The aim of the following analysis was to present the treatment outcomes of less invasive surfactant administration technique (LISA), which was recently implemented into daily practice in treating preterm infants, in relation to the results obtained in the group of neonates receiving surfactant with the use of the INSURE method, which has been applied since 1999.

## Material and methods

**Fig. 1:**
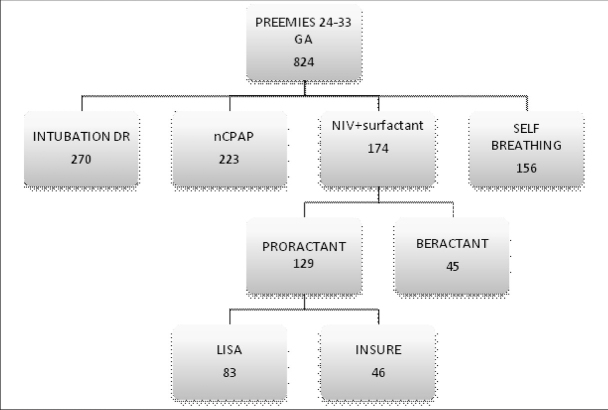
The structure of the population of all preterm infants born at a gestational age between 24 and 33 weeks who were hospitalized in the Neonatology Clinic in the years 2014-2016. The two final study groups are: LISA (n=83) and INSURE (n=46). Ryc. 1. Diagram prezentujący analizowaną grupę noworodków na tle wszystkich hospitalizowanych wcześniaków o dojrzałości 24-33 tyg. ciąży w latach 2014-2016. Ostateczne grupy: LISA (n-83), INSURE (n-46).

Out of the 824 infants born at a gestational age of between 24 and 33 weeks and hospitalized in our Neonatology Department in the years 2014-2016, 270 were intubated already in the delivery room or within the first hour of life, 223 required only nCPAP respiratory support without the need for surfactant administration, 156 did not need either respiratory support or surfactant administration, and 174 received surfactant (proractant or beractant) with the use of a non-invasive method (LISA or INSURE). Newborns with severe congenital disorders were excluded from the study. The retrospective analysis included 129 infants born at a gestational age of between 24 (24 + 0/7) and 33 (32 + 6/7) weeks who required surfactant administration due to respiratory distress syndrome [4]. Only proractant alfa was administered with the use of either the LISA or the INSURE technique and the choice of the method was made by the physician in charge. In total 83 neonates received surfactant via LISA and 46 neonates via INSURE. (Fig. 1).

The infants in both study groups received a median dose of 200 mg/kg of proractant alfa (median dose in LISA group: 200 mg/kg [min. 115 mg/kg; max. 250 mg/ kg]; median dose in INSURE group: 187 mg/kg [min. 134 mg/kg; max. 230 mg/kg]; p<0.001). The clinical data provided in the database included: the respiratory support method and the fraction of inspired oxygen 3, 6, 12, 24, 48 and 72 hours after surfactant administration; the need for intubation within the first 48 hours after surfactant administration; the need for the second and subsequent surfactant doses; the duration of mechanical ventilation, nCPAP and oxygen supplementation. The incidence of complications (pneumothorax and pulmonary hemorrhage) was also analyzed. Moreover, both groups were compared in terms of the presence of typical complications of prematurity: intraventricular hemorrhage (IVH), patent ductus arteriosus (PDA), retinopathy of prematurity (ROP), necrotizing enterocolitis (NEC), bronchopulmonary dysplasia (BPD).

The second and subsequent doses of surfactant were given to those infants who experienced only temporary respiratory improvement after the first dose, who presented radiological evidence of ongoing respiratory distress syndrome and who fulfilled the criteria for second dose administration in the subsequent hours of life [3]. IVH diagnosis was made based on ultrasonography (Philips HD11 XE) examination using the Papile classification [4]. The diagnosis of PDA was based on the clinical picture and echocardiographic evaluation of hemodynamic parameters. ROP was diagnosed by a consulting ophthalmologist using standard classification [5], and NEC by means of using the Bell classification [6]. The criteria for congenital infection included positive blood or cerebro-spinal fluid cultures or clinical features of infection with elevated inflammatory markers. Histological examination of the placenta, as well as the data on maternal colonization with pathological microbial flora were also taken into consideration in diagnostic decision-making. The study used the definition of BPD that was established in 2000 by the National Institute of Child Health and Human Development (NICHD). Mild BPD was diagnosed in neonates who required supplemental oxygen use for 28 days of their life. Moderate and severe BPD was diagnosed in infants who needed <30% oxygen at 36 weeks’ postmenstrual age or >30% oxygen /continuous positive pressure ventilation at 36 weeks’ postmenstrual age, respectively. Nominal variables were presented as numbers with percentages and analyzed using the chi-square test with appropriate corrections (the Yates’s correction for continuity or the Fisher exact test), if needed. The normality of the distribution of continuous variables was verified with the Shapiro-Wilk test. Continuous variables were presented as medians with 25% to 75% values and compared using the Mann-Whitney U test. Paired comparisons across the time points were analyzed using repeated measures of the analysis of variance. Multivariable analysis was performed using general linear models. The statistical analysis was done using Statistica 13.1 software (Statsoft, Poland). P values lower than 0.05 were considered statistically significant.

## Results

There were no significant differences in patient characteristics between the LISA and INSURE study groups. A high incidence of prenatal steroids (over 90% in both groups) and a high prevalence of congenital infection (54.2% LISA vs 69.6% INSURE) was observed in the whole population (Tab. I).

**Table I:** Study population characteristics. Tabela I. Charakterystyka badanej populacji.

	LISA (N=83)	INSURE (N=46)	p value
Gestational Age, weeks *Wiek płodowy, tygodnie*	30.0	29 6/7	0.503
Median (25-75%)	(28 1/7-31 6/7)	(28 5/7- 30 6/7)	
Birth Body Weight, g *Masa urodzeniowa, g* Median (25-75%)	1210 (1000-1700)	1275 (930-1600)	0.811
1 min Apgar score *Apgar w 1 min* Median (25-75%)	6 (6-7)	7 (6-7)	0.331
5 min Apgar score *Apgar w 5 min* Median (25-75%)	7 (6-8)	7 (7-8)	0.154
Sex; *Płeć* Female, N(%); *Żeńska*	42 (50.6)	23 (50.0)	0.948
Male, N(%); *Męska*	41 (49.4)	23 (50.0)	
Method of delivery *Sposób rozwiązania ciąży* Vaginal delivery, N (%) *PSN*	15 (18.0)	3 (6,5)	0.011
Cesarean section, N (%) *Cięcie cesarskie*	68 (52.0)	43 (93,5)	
Antenatal steroids, N (%) *Steroidy prenatalnie*	75 (90.36)	44 (95.65)	0.493
Congenital infection, N(%) *Infekcja wrodzona*	45 (54.2)	32 (69.6)	0.086

The comparison of early results of respiratory failure treatment did not reveal any significant differences between the two groups in terms of the need for intubation within the first 48 hours after surfactant administration, the need for subsequent surfactant doses, and the incidence of pulmonary hemorrhage. Although pneumothorax was observed in 7.2% and 2.2% of the LISA and INSURE patients, respectively, the difference did not prove to be statistically significant. Similarly, the analysis of long-term treatment outcomes (the number of days on mechanical ventilation, the number of days requiring nCPAP and oxygen supplementation) showed no differences between both study groups (Tab. II).

**Fig. 2:**
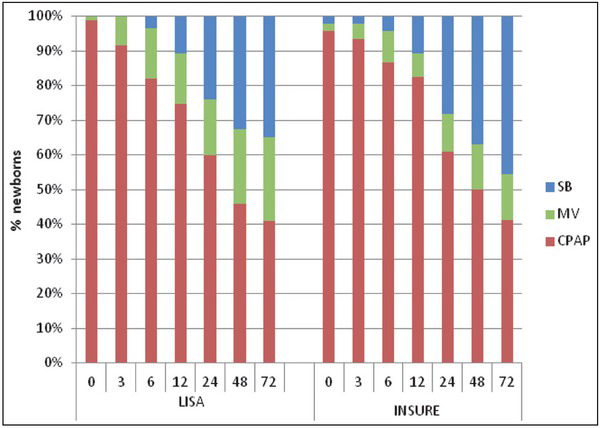
The percentage of neonates on spontaneous breathing (SB), mechanical ventilation (MV) or CPAP in relation to time from surfactant administration. Ryc. 2. Procent noworodków pozostających na oddechu własnym (SB), wentylacji mechanicznej (MV) oraz CPAP przedstawiony w funkcji czasu, od momentu podania surfaktantu (0).

In order to determine the most effective method, a detailed analysis of the first 72 hours after surfactant administration was performed. Respiratory support methods (mechanical ventilation, nCPAP, spontaneous breath), as well as oxygen demand, were evaluated at subsequent timepoints (3, 6, 12, 24, 48 and 72 hours after surfactant administration) in both study groups. Although no statistically significant differences were shown, a tendency for faster respiratory improvement (faster respiratory support withdrawal, faster reduction of FiO_2_ concentration) in the INSURE group was observed (Fig. 2).

**Fig. 3:**
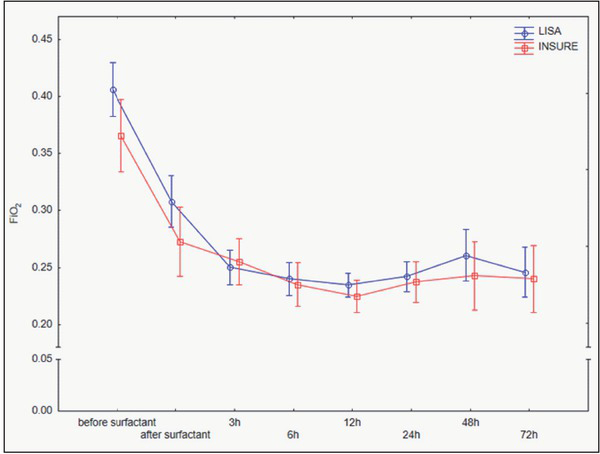
Interaction between FiO_2_ % and time in LISA and INSURE group (p=0.193). The vertical lines represent a 95% confidence interval. Ryc. 3. Zależność FiO_2_ % w funkcji czasu w grupach LISA oraz INSURE (p=0.193). Pionowe linie reprezentują 95% przedziały ufności.

One patient from each group required intubation within the first hour from surfactant administration due to clinical features of respiratory distress. All the patients experienced fast clinical improvement independent of the surfactant administration method, and the reduction of FiO_2_ concentration was most dynamic within the first 3 hours (Fig. 3).

The incidence of complications was similar in both study groups (Tab. III).

**Table II:** Respiratory outcomes in neonates born between 24 and 33 weeks of gestation, LISA and INSURE. Tabela II. Wyniki leczenia niewydolności oddechowej noworodków o dojrzałości 24-33 tyg. ciąży w grupach LISA oraz INSURE.

	LISA (N=83)	INSURE (N=46)	p value
Intubation 48h after surfactant administraon, N (%) *Intubacja w ciągu 48 h od podania surfaktant*	35 (42.2)	15 (32.6)	0.201
Surfactant II dose, N (%) *II dawka surfaktantu*	8 (9.6)	4 (8.7)	0.642
Pneumothorax, N (%) *Odma opłucnowa*	6 (7.2)	1 (2.2)	0.42
Pulmonary hemorrhage, N (%) *Krwotok płucny*	4 (4.8)	1 (2.2)	0.654
Mechanical ventilation, days *Wentylacja mechaniczna, dni* Median (25-75%)	0 (0-3)	0 (0-2)	0.377
CPAP, days *CPAP, dni* Median (25-75%)	5 (2-16)	5 (2-11)	0.379
Oxygen supplementation, days *Tlenoterapia bierna, dni* Median (25-75%)	1 (0-9)	1 (0-11)	0.555

## Discussion

Strong emphasis has been placed on the non-invasive respiratory support methods as techniques of choice in preterm neonates over the last decade. Moreover, great efforts have been made to find an optimal way of surfactant administration. Recent studies suggest that the best approach in preterm infants who require surfactant administration during non-invasive respiratory support is the LISA method. However, our results did not prove the superiority of LISA over INSURE. Neither LISA nor INSURE were associated with better respiratory outcomes in neonates born between 24 and 33 weeks of gestation.

A randomized study by A. Kribs [7] included less mature infants (born under 31 weeks of gestation) and compared the LISA group with newborns receiving standard therapy, of whom only 47% were treated with surfactant. A significant reduction in the need for mechanical ventilation within the first 72 hours (29% *vs* 53%, respectively; p<0.001) and a decrease in BPD incidence (10.9% *vs* 17.5%, respectively; p=0.004), as well as in the total number of BPD cases and deaths, were observed in the LISA groups when compared with standard care patients.

**Table III:** Premature birth complications in the LISA and INSURE study groups, neonates born between 24 and 33 week of gestation. Tabela III. Powikłania wcześniactwa w grupach LISA oraz INSURE u noworodków o dojrzałości 24-33 tyg. ciąży.

	LISA (N=83) No. (%)	INSURE (N=46) No. (%)	p value
BPD all grades *BPD wszystkie stopnie*	24 (28.9)	11(23.9)	
BPD mild *BPD łagodna*	12 (14.5)	7 (15.2)	
BPD moderate *BPD* *umiarkowana*	7 (8.4)	1 (2.2)	0.506
BPD severe *BPD* *ciężka*	5 (6.0)	3 (6.5)	

IVH all grades *IVH wszystkie stopnie*	22 (26.5)	13 (28.2)	
IVH grade III/IV *IVH stopień III/IV*	2 (2.4)	0 (0)	0.562

ROP all grades *ROP wszystkie stopnie*	11(13.2)	3 (6.5)	0.272
ROP (laser therapy) *ROP (fotokoagulacja laserowa)*	8 (9.6)	1 (2.2)	

PDA hs *PDA* *istotne hemodynamicznie* PDA (pharmacological treatment) PDA *(leczenie farmakologiczne)*	28 (33.7) 23 (27.7)	15 (33.3) 14 (31.1)	0.568
PDA (ligation) *PDA* *(ligacja)*	5 (6.0)	1 (2.2)	

NEC all grades *NEC* *wszystkie stopnie*	3 (3.6)	1 (2.2)	
NEC ** (surgical *NEC (leczenie* treatment) *chirurgiczne)*	1 (1.2)	1 (2.2)	0.379

Death *Zgon*	4 (4.8)	2 (4.4)	1.0

To date, only 3 studies comparing the LISA and INSURE methods have been published [8, 9, 10]. In a study by Kanmaz [8] including newborns of similar age (born under 32 weeks of gestation), a clear improvement of respiratory outcomes was observed in the LISA group: the need for mechanical ventilation was lower (30% *vs* 45%, respectively; p=0.02), while mean times of nCPAP and mechanical ventilation significantly shorter (p values 0.006 and 0.002, respectively) as compared with the INSURE group. What is more, the incidence of BPD was lower in the LISA group and the difference proved to be statistically significant (relative risk 0.27). Although the results of the randomized trials by Bao [9] and Mohammadizadeh [10] support the superiority of the LISA method, the conclusions are not unequivocal. In a study conducted by Chinese investigators [9], it has been observed that the LISA method can be safely applied in neonates born between 24 and 33 weeks of gestation and that it helps to reduce the time of mechanical ventilation and nCPAP (presented as a common end point). However, it did not influence the incidence of BPD. On the other hand, the results of the Iranian study [10] did not show any statistically significant differences between the LISA and INSURE methods, except for the correlation between LISA and oxygen supplementation time.

The size of our study population was small (n=129). A higher sample size might probably have increased the significance level of the findings. According to the current literature, the LISA method is more efficient compared to other non-invasive strategies (e.g. INSURE method). However, this has been proven mainly through meta-analyses [11, 12], which included the results from multiple centers. Optimal sampling seems to be of great importance for the discussion of LISA and INSURE benefits, as both of the techniques may have a positive and negative impact on the youngest preemies.

While considering the efficacy of the LISA method, one should take account of the fact that the infant stays on non-invasive respiratory support during the whole procedure. For that reason, the nCPAP interface (nasal prongs, mask, single nasal catheters) must be kept in the child’s nostrils. Depending on its size, the interface may hinder the laryngoscopy and introduction of a thin catheter into the trachea. Moreover, maneuvers during the LISA procedure can lead to the displacement of nCPAP. As a consequence, positive end-expiratory pressure is no longer delivered to the infant’s airways. The catheter itself may also be a cause of inconvenience (it can be too flexible, too long, sharp-ended, or have no scale). Various catheters are being used during the LISA procedure (e.g. umbilical catheters, feeding tubes, suction catheters, vascular catheters) [13]. While introducing the LISA method in our center, we were using either Angiocath vascular catheters (accompanied by a guideway due to the insufficient rigidity of the catheter) or Impress catheters for intravenous contrast injections (rigid enough, but too long). Since 2018, catheters dedicated to the LISA method have been available, so the delivery of surfactant has become much more convenient.

A common side effect of surfactant administration with the LISA method is surfactant reflux. It is probably caused by the fact that the thin catheter does not fully fill the larynx and surfactant can spontaneously reflux to the oral cavity. The results of a study performed on animal models (lambs) by Niemarkt [14] showed that blood oxygenation in lambs who received surfactant using the LISA method was similar to blood oxygenation in those who received surfactant after intubation. In the lambs that received LISA, lung compliance was lower, and the amount of the surfactant found in the lungs was only 17.4±0.8% compared to the amount of surfactant in the lungs of the lambs that were intubated (p<0.05). In the study on rabbit models conducted by Bohlin [15], the animals were treated with pharyngeal deposition of surfactant and then randomized to get mechanical ventilation or spontaneous breathing. It has been observed that the lung compliance and distribution of surfactant were higher in the spontaneously breathing group. These results seem to be an additional argument in favor of choosing the LISA method in preterm infants with preserved spontaneous breath and limiting the use of mechanical ventilation only to situations when it is really required. The application of the INSURE method does not allow to completely avoid mechanical ventilation and poses a potential risk of iatrogenic laryngeal or tracheal damage. Moreover, intubation induces neonatal trauma. During INSURE, the infant is at risk for lung damage due to invasive ventilation, especially if ventilation time is not strictly defined. INSURE can be limited to 15 minutes or last from 30-60 minutes up to several hours. In our group, the mean duration of mechanical ventilation in the INSURE group was approximately 10-15 minutes.

What remains controversial is the problem of sedation. Tracheal laryngoscopy is associated with pain and discomfort. The use of sedatives during LISA is arbitrary [13], and many experts do not apply sedation, making it possible to maintain regular, spontaneous breath during the whole procedure. For that reason, the use of sedatives must be considered on an individual basis, and the team should be well-trained.

The LISA method is becoming increasingly popular in neonatology departments. Meta-analyses [11, 12] that have been published recently, prove its superiority over the INSURE procedure in terms of limiting respiratory complications in preterm infants. A meta-analysis [11] conducted by Canadian researchers investigated different treatment approaches for respiratory distress in neonates born under 33 weeks of gestation. All of the following: nCPAP, INSURE, LISA, noninvasive intermittent positive pressure ventilation (NIPPV), surfactant administration through nebulization or laryngeal mask and classical mechanical ventilation were compared. Only 3 out of the 30 studies that were included provided a direct comparison of LISA and INSURE [7, 8, 9]. The results of the meta-analysis showed that the LISA method was associated with the lowest risk of BPD development or death, as well as severe IVH, as compared with mechanical ventilation. Taken together, LISA and INSURE pose a low risk for BPD or death compared to mechanical ventilation and a lower risk of pneumothorax compared to the nCPAP approach. The LISA method constituted the best treatment strategy that makes it possible to maintain spontaneous breath in preterm neonates requiring surfactant administration. The second best method was INSURE. Finally, the meta-analysis published in Global Pediatric Health in 2016 [12] included only 3 randomized trials and directly compared LISA to the INSURE approach. A significant reduction in the need for mechanical ventilation and its duration during the first 72 hours, as well as in oxygen supplementation and nCPAP support was observed in the LISA group. A tendency for decreased BPD incidence without an influence on survival rates has also been reported. The study by Rigo [16] confirmed that the risk of death, BPD incidence, and nCPAP failure are lower in LISA compared to INSURE group.

Of note, the study period between 2014 and 2016 was also the time of learning and mastering the LISA technique in our center. The initial absence of an optimal, LISA-dedicated catheter, as well as the quest for the proper nCPAP interface influenced the final study outcomes and might have contributed to the lack of sufficient evidence for the superiority of the LISA approach in terms of safety and benefits for preterm neonates in the first years of use.

## Conclusions

The analysis of the initial results of surfactant administration with the use of the LISA method did not confirm the superiority of LISA over INSURE. Our observations suggest that both techniques are comparable. Reliable findings can be obtained if all the elements influencing the final effect of surfactant administration with non-invasive support are refined (team skills, choice of the equipment). Further randomized trials are needed to establish the best respiratory approach in preterm infants requiring surfactant administration. The randomized, prospective study that is currently being conducted in the Neonatology Department and includes biochemical markers of lung damage, will bring more objective data on the safety and effectiveness of both surfactant administration techniques (LISA *vs* INSURE).
